# Endocrine Disruptors in Child Obesity and Related Disorders: Early Critical Windows of Exposure

**DOI:** 10.1007/s13668-024-00604-1

**Published:** 2025-01-08

**Authors:** Mensure Nur Celik, Ozge Yesildemir

**Affiliations:** 1 Department of Nutrition and Dietetics, Faculty of Health Sciences, Ondokuz Mayıs University, Samsun, Turkey; 2 Department of Nutrition and Dietetics, Faculty of Health Sciences, Bursa Uludag University, Bursa, Turkey

**Keywords:** Endocrine disruptors, Prenatal exposure, Postnatal exposure, Obesity, Diabetes, Metabolic Disorders

## Abstract

**Purpose of Review:**

Endocrine disruptors (EDs) can mimic or interfere with hormones in the body, leading to non-communicable diseases, such as obesity, diabetes, and metabolic syndrome. Susceptibility to EDs increases during prenatal and postnatal life, a critical time window. This review aims to summarize the latest evidence on the relation of early life exposure to some EDs with obesity and the other metabolic disorders.

**Recent Findings:**

There is increasing evidence that early life exposure to EDs may impair adipogenesis by increasing the number and size of adipocytes, thereby increasing susceptibility to obesity in childhood. It is stated that exposure to EDs during the prenatal and postnatal period may raise the risk of type 2 diabetes in adulthood by disrupting glucose, lipid, and insulin homeostasis in the offspring. They can also accelerate the development of type 1 diabetes through various mechanisms, like immunomodulation, gut microbiota, and vitamin D pathways.

**Summary:**

There is a growing understanding that ED exposure during critical stages of life could play an important role in the development of obesity and metabolic disorders. We suggest setting national goals, global standards, and policies to reduce environmental exposure to pregnant and lactating women, and babies, considered sensitive populations.

## Introduction

Natural and synthetic chemicals can imitate or disrupt the endocrine system, a complex communication network that connects the nervous system with vital biological functions [1]. Endocrine disruptors (EDs) are chemicals present in many everyday items like food packaging, detergents, toys, and cosmetics. Their widespread presence makes it crucial for scientists to investigate how they might act as risk factors or triggers for health issues.[2].

There is increasing evidence that EDs are serious threats to human health [3]. The 2015 Parma Consensus emphasized that endocrine disruptors can interfere with metabolic systems during crucial developmental periods, potentially contributing to non-communicable diseases like obesity, diabetes, and metabolic syndrome [4]. Additionally, prenatal exposure to EDs, even at low concentrations, has been noted to have an impact on cardiometabolic risk factors in children [5].

Pregnancy is a particularly vulnerable time for both the mother and the developing embryo, as the effects of endocrine disruptors can affect both simultaneously. At the same time, newborns can be exposed to EDs during the postnatal period through breastfeeding or infant formula feeding. The literature contains conflicting data regarding the immediate and long-term effects of exposure to endocrine disruptors on mothers, fetuses, and newborns [6]. The impact of environmental pollutants on children's health is significantly influenced by when they are exposed [7]. In line with this information, this article aims to review the latest findings on exposure to EDs, focusing on the effects of prenatal and postnatal exposure to EDs in children with metabolic disorders.

## Metabolic Diseases

There is consensus that EDs may disrupt glucose and lipid metabolism and contribute to the worldwide spread of obesity and metabolic disorders [3, 8]. Some EDCs (for example; BPA, phthalates, PCBs, DDT, DDE, PFAS, dioxins and furans examined in this review) can be transferred to the fetus via the placenta during pregnancy in humans [9]. Additionally, breastfeeding significantly contributes to early life exposure [10]. "Metabolism-disrupting chemicals (MDCs)" were identified in 2017 as a subgroup of endocrine disruptors that impair metabolic functions and may contribute to diseases like obesity and type 2 diabetes [11]. These chemicals can affect energy homeostasis by altering the differentiation and function of white adipose tissue and changing serum levels of insulin, leptin, and fatty acids [11].

Obesogens can interfere with adipocyte balance by interacting with receptors like peroxisome proliferator-activated receptor gamma (PPARγ) and 9-cis retinoic acid receptor (RXR). This can lead to a higher number and larger size of fat cells, impaired glucose uptake and insulin signaling, decreased expression of brown fat markers, and increased inflammation [12]. Obesogens may contribute to obesity by affecting metabolic rate, energy balance, and the hypothalamic control of hunger and fullness [13]. They can also mimic natural estrogens by binding to estrogen receptors (ERα and ERβ) and disrupt the endocrine system by interfering with androgen receptors (ARs) and thyroid hormone receptors (TRs). [14–16]. The mechanisms behind these metabolic disorders are explored in more detail below.

### Obesity

Understanding obesity has traditionally focused on diet and lack of physical activity, but there is increasing concern about how exposure to endocrine disruptors might affect growth during fetal development and childhood [17].

**Fig. 1:**
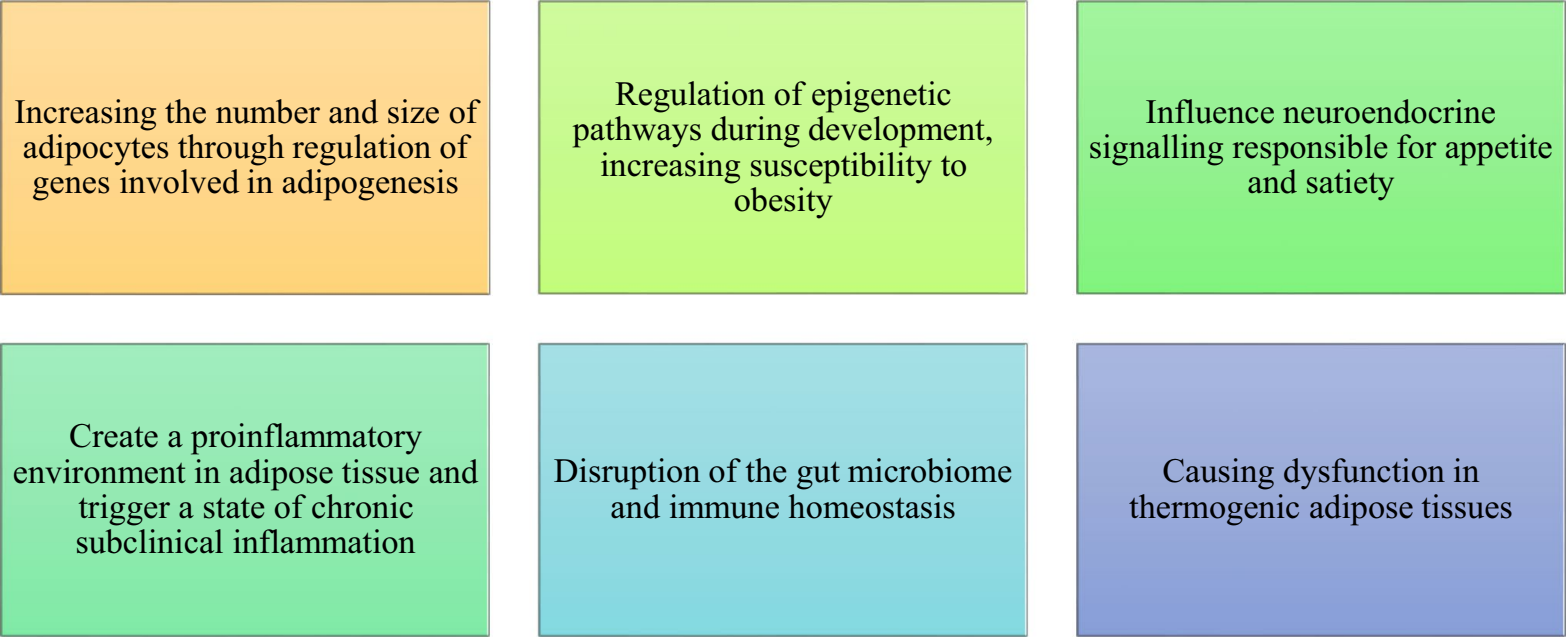
Proposed mechanisms of action of endocrine disruptors on obesity

The environmental obesogen hypothesis, proposed in the early 2000s, suggests that certain synthetic chemicals, known as obesogens, may increase the risk of obesity and metabolic diseases [18, 19]. The rise in chemical production, alongside the growing rates of overweight and obesity, has prompted numerous studies to explore a possible causal relationship between these factors [19]. According to the "environmental obesogen hypothesis" put forward in this context; It has been shown that exposure to EDs early in life (including in utero) may impair adipogenesis or energy storage-related mechanisms by increasing the number/size of adipocytes, thereby increasing susceptibility to overweight/obesity [3, 18]. The mechanisms by which “obesogenic” EDs contribute to the etiology of obesity are as follows: direct promotion of adipogenesis, which increases both the number and size of adipocytes through activation of PPARγ, increased differentiation of preadipocytes towards adipose tissue through activation of PPARγ, increased fat, which promotes activation of the transcription factor of adipogenic genes. accumulation and promotion of potential epigenetic mechanisms. Many EDs accumulate in adipose tissue, leading to interactions and changes in endocrine activity and homeostatic systems underlying body weight control [8, 20]. A recent review, based on available data from mechanistic, animal, and epidemiological studies, including meta-analyses, suggests that EDCs contribute to the development of obesity, related disorders, and obesity-related adipose tissue dysfunction by affecting adipogenesis, increasing susceptibility to obesity by regulating epigenetic pathways during development, affecting neuroendocrine signals responsible for appetite and satiety, promoting a proinflammatory environment in adipose tissue and inducing a chronic subclinical inflammatory state, disrupting the gut microbiome and immune homeostasis and inducing dysfunction in thermogenic adipose tissue [21]. Figure 1 summarizes the general mechanisms of action of endocrine disruptors on obesity.

The most studied EDs in epidemiological studies are since there are bisphenol A (BPA), phthalates, and persistent organic pollutants (POP) [22]. These topics are given in detail below.

#### Bisphenol A (BPA)

The detection of BPA in blood, urine, adipose tissue, breast milk, placental tissue, and amniotic fluid indicates that exposure begins prenatally [23]. BPA, known as an endocrine disruptor, can mimic hormone activity through estrogen and PPARγ signaling and is considered an obesogen that promotes adipogenesis [24–26]. While BPA is discussed in relation to increasing the risk of obesity, studies on human prenatal BPA exposure are limited and inconsistent [27–29]. Although more animal studies are available, they also show inconsistent results, and the mechanisms of BPA-related epigenetic changes are not well understood. There is no data on BPA-induced epigenetic modifications that contribute to overweight development [30]. One study found that BPA levels in urine samples from pregnant women (6.3–15 weeks) were associated with their children's body weight, height, waist/hip circumference, and skinfold thickness at ages 1.9–6.2 years. These findings suggest a positive association between gestational urinary BPA levels and central adiposity in young girls [31].

A recent systematic review suggests that prenatal BPA exposure may increase the risk of obesity in offspring, with differences observed between sexes. This underscores the need for expectant mothers to reduce or avoid BPA exposure during pregnancy. However, the review notes significant variability and inconsistencies across studies, which complicate the interpretation of the relationship between prenatal BPA exposure and offspring obesity. Further research is required to clarify the causal connection between prenatal BPA exposure and obesity at different developmental stages and genders, and to understand the underlying mechanisms [32].

Epidemiological studies investigating the connection between BPA exposure and childhood overweight or obesity have yielded inconclusive results. However, cross-sectional studies often find a positive association between higher urinary BPA levels and obesity [33–37].

In several prospective cohort studies, higher prenatal BPA exposure has been associated with higher body mass index (BMI), or body weight for height, among children [27, 38], while in some studies, higher exposure in early childhood has been associated with lower BMI [28, 29]. Urinary BPA concentrations were more strongly linked to an increased risk of general and abdominal obesity in boys compared to girls aged 6–17 years, indicating a potential sex-related difference [39]. Studies on the relationship between BPA concentrations and obesity in children have yielded mixed results. Some research suggests that higher BPA levels in children's urine are associated with increased BMI at age 4, with prenatal BPA exposure correlating negatively with BMI in girls and positively in boys. However, the findings are controversial, as other studies report no significant link between urinary BPA levels and obesity, whether general or abdominal [40–42].

#### Phthalates

Phthalates, like BPA, can contribute to obesity through similar mechanisms. They affect energy homeostasis by influencing nuclear receptors such as Erα, Erβ, PPARγ, TRs, and ARs, increasing the risk of obesity [43]. Experimental studies suggest that exposure to phthalates during pregnancy and childhood can impact body composition through several mechanisms [4, 44]:

- Some phthalates exhibit weak estrogenic activity and strong anti-androgenic effects, potentially disrupting sex-specific growth patterns [45].
- Phthalates can alter PPARs, which are crucial for regulating adipocyte differentiation, inflammation, and lipid and carbohydrate metabolism [46–48].
- They may also affect adipokine production, impacting metabolic functions like appetite regulation, thermogenesis, fatty acid oxidation, and glucose levels. These disruptions can lead to inflammation and endothelial dysfunction, affecting body composition during adolescence and into adulthood [48].
- Phthalates are associated with reduced thyroid hormone levels, which can disrupt hormonal balance and contribute to fat accumulation and obesity [49].

Some epidemiological studies suggest a link between prenatal or postnatal phthalate exposure and child adiposity, but the findings are inconsistent [50–53]. Most of these studies are cross-sectional, and the associations between phthalate exposure and adiposity markers can differ depending on factors like the study population, sample size, timing, and level of exposure [54]. These inconsistencies make it difficult to draw definitive conclusions.

Phthalate metabolites are nearly always detectable in the urine of pregnant women [55]. They can also be found in amniotic fluid and placental tissue, indicating that these metabolites transfer to the fetüs [56, 57]. During fetal development, these metabolites can influence molecular mechanisms and epigenetic processes, potentially affecting metabolism and adipogenesis throughout an individual's life [58]. Therefore, exposure to environmental chemicals in utero may be the most critical window period for human health. In analyzes of prospective cohort studies, a sex-specific (negative in girls and positive in boys) association between prenatal (during pregnancy) monoethyl phthalate (MEP, i.e., DEP metabolite) exposure and BMI at ages 4–7 was reported [59]. Some studies (in rats) report a significant association between prenatal phthalate exposure and reduced BMI z-scores, but not with fat mass index. This may be explained by the antiandrogenic effects of phthalates, which could impact muscle development [45, 60]. Epidemiological evidence supports that androgens are linked to muscle growth [61, 62]. In a cross-sectional study, increased urinary concentration of phthalate metabolites was shown to be associated with decreased lean body mass [63]. In a cross-sectional study, increased urinary concentration of phthalate metabolites was shown to be associated with decreased lean body mass [64].

In a cross-sectional study, increased urinary concentration of phthalate metabolites was shown to be associated with decreased lean body mass [53, 65]. Human/animal studies indicate that PPARs, which are nuclear hormone receptors involved in regulating adipogenesis and lipid storage, play a crucial role in promoting adipogenesis and obesity [24, 46, 66]. Research on the link between phthalates and higher BMI suggests that increased energy intake in overweight and obese individuals may lead to higher phthalate exposure [67].

Consumption of ultra-processed foods is linked to overweight and weight gain and has also been associated with higher levels of phthalate metabolites in urine [68, 69]. Thus, the observed connection between phthalate metabolites and obesity may be influenced by dietary patterns and food intake. Furthermore, urinary phthalate metabolites may be higher in children with greater fat and muscle mass [64].

A recent systematic review consistently found a positive association between prenatal exposure to most phthalates and obesity in offspring. However, studies on postnatal phthalate exposure have shown mixed results, with both positive and negative associations observed. These conflicting findings might be due to differences in study design, and other lifestyle factors, such as diet and physical activity after birth, could also influence the risk of obesity [70].

#### Persistent Organic Pollutants (POPs)

POPs may contribute to obesity by increasing the number of fat cells, enhancing fat storage, altering the basal metabolic rate, shifting energy balance towards calorie storage, and disrupting the hormonal regulation of appetite and satiety [11, 17, 44].

POPs, or organochlorine compounds, have been suspected of acting as obesogens. Key compounds like PCBs, DDT and its metabolite DDE, and HCB are frequently studied in birth cohort studies. These lipophilic compounds can bioaccumulate in human tissues [71]. A meta-analysis found that prenatal exposure to DDE and HCB is associated with increased BMI in children, but there is no clear evidence linking prenatal exposure to PCBs, PFAS, or PBDEs with childhood obesity [72].

Maternal POP concentrations can transfer to the developing fetus through the placenta [73]. During the intrauterine period, the fetus is particularly vulnerable due to rapid cellular differentiation and tissue development, along with underdeveloped protective mechanisms like xenobiotic metabolism, immune function, and the blood–brain barrier [11, 17]. POPs are widely studied as potential obesogens in early life exposures. Numerous epidemiological studies have examined the link between early exposure to POPs (in utero and during childhood) and the risk of childhood obesity. Most research focuses on single indicators like BMI or body weight, with fewer studies investigating other adiposity measures like waist circumference, waist-to-height ratio, or skinfold thickness [74–78]. In this section, POPs such as PCBs, DDT, DDE, HCB, PFAS-PFOS, dioxins and furans are mentioned in relation to child growth.

##### Polychlorinated biphenyls (PCB)

Possible mechanisms of action proposed to explain the association of PCB exposure with higher body weight include:

- PCBs interact with PPARγ and Retinoid X Receptor (RXR), which are crucial for adipogenesis, lipid storage, and regulating adipocytokines [79, 80],
- Anti-androgenic and xeno-estrogenic effects by disrupting endocrine signaling, interfering with the hypothalamus and pituitary-thyroid axis, inhibiting androgen receptors, enhancing estrogen activity, or reducing peripheral androgen conversion [81, 82],
- Exposure to PCBs has been associated with alterations in cytokine production and lipid and glucose metabolism, which can lead to weight gain due to interactions with the gut microbiota, as suggested by animal studies [83].

While it has been reported that the findings regarding the relationship between prenatal PCB exposure and childhood obesity are not consistent [84], an analysis of 7 birth cohorts in Europe reported that postnatal PCB exposure was associated with reduced infant growth [85]. However, another study found no identified negative association of prenatal exposure to PCBs with low birth weight [86].

##### Dichlorodiphenyl trichloroethane (DDT) and Dichlorodiphenyl dichloroethene (DDE) 

A review found inconsistent results regarding the relationship between DDT and DDE exposure and birth weightSome studies found a negative association between DDT levels and birth weight, while others reported no link between DDT or DDE exposure and birth weight [87]. Additionally, a meta-analysis of 12 European birth cohorts found that the association between cord serum DDE levels and birth weight was not statistically significant [88]. In several birth cohort studies, prenatal exposure to DDT and DDE was found to be positively associated with BMI during infancy or childhood [75, 89–93].

No association was found between postnatal p,p0-DDE exposure and weight gain during childhood or adolescence [94]. However, postnatal DDE exposure showed a significant association with other obesity-related indicators, such as leptin concentration in the serum of adolescent males [95].

##### Hexachlorobenzene (HCB)

 A review of the literature reports that there is an inconsistent relationship between exposure to HCB during pregnancy and the risk of obesity [96]. While various studies have reported a negative relationship between HCB levels and birth weight [97–99], many studies have revealed that there is no relationship between the level of prenatal exposure to HCB and birth weight [100–102].

##### Per- and polyfluoroalkyl substances (PFAS)

 The most common PFAS, PFOA and PFOS, are persistent in the environment due to their resistance to degradation [103]. systematic review found that higher maternal levels of PFOA or PFOS are linked to lower birth weight, length, and ponderal index [104]. One study reported a negative association between early maternal PFOA or PFOS levels and postnatal growth (weight and BMI) at 5 and 12 months, though later results showed no link to BMI or waist circumference at 7 years [105, 106]. Another study found a positive association between prenatal PFOS exposure and infant weight at 20 months [107].

#####  Dioxins and furans

 A small meta-analysis of two cohort studies found a higher risk of low birth weight associated with prenatal dioxin exposure [108]. Another analysis of three European birth cohorts linked prenatal exposure to dioxins, furans, and dioxin-like compounds with increased weight gain from 0 to 24 months. By age seven, dioxin exposure was significantly linked to increased BMI in girls, with a 54% higher risk of becoming overweight over seven years, but no significant effect was observed in boys [109].

### Diabetes Mellitus

The pancreas is one of the most important targets of ED toxicity. When exposed to EDs, pancreatic β cells can undergo critical impairment in insulin production and secretion [110]. Some endocrine disruptors can directly induce insulin resistance and impair insulin production and secretion without significantly impacting body weight. They can disrupt glucose homeostasis by affecting the cells responsible for secreting both insulin and glucagon [111]. The effects of endocrine disruptors on glucose metabolism in children are given in Table 1.

**Table 1:** Effects of endocrine disruptors on glucose metabolism in children

Endocrine Disruptors	Health Outcomes	Potential Mechanisms
Bisphenol A	• Disturbance of glucose homeostasis and normal physiology of pancreatic cells • Impairment of biosynthesis and secretion of insulin in β cells	• Binding to estrogenic receptors (Erα and Erβ) and androgen receptors (ARs)
Phthalates	• Causing adipogenesis, lipid accumulation and insulin resistance • Disturbance in glucose homeostasis • Inducing insulin resistance and hepatic lipid accumulation	• Thyroid hormone and androgen antagonist • Affecting the JAK2/STAT3/SOCS3 pathway, which is involved in the regulation of insulin and leptin signaling pathways • Overexpression of FOXO1
Persistent organic pollutants	• Insulin resistance • Glucose dysregulation • Prediabetes/diabetes	• Interference with PPAR expression • Anti-androgenic and anti-estrogenic effect • Impaired β-cell insulin secretion • Affecting insulin signaling in metabolically active tissues
Organophosphorus pesticides	• Dysregulation of glucose tolerance and insulin sensitivity	• Disturbance in glucose and lipid metabolism

ERα Estrogen receptor alpha and ERβ; estrogen receptor beta; FOXO Forkhead Box O1; PPARγ Peroxisome proliferator-activated receptor gamma.

Chemicals that damage or disrupt pancreatic β cells are termed "diabetogenic." These substances can directly induce insulin resistance and impair insulin production and secretion. According to the "diabetogenic hypothesis," any endocrine disruptor in the bloodstream that causes insulin resistance may be a risk factor for metabolic syndrome and Type 2 Diabetes Mellitus (T2DM), regardless of its potential to cause obesity or accumulate in adipose tissue [111]. Human studies on the effects of endocrine disruptors (EDs) on the development of Type 1 Diabetes Mellitus (T1DM) are controversial and require further investigation, especially given the rising incidence of T1DM globally [112]. PFASs, in particular, may trigger autoimmune processes affecting β cells in T1DM [113]. High prenatal exposure to PFASs can alter lipid profiles in newborns and potentially increase the risk of islet autoimmunity and T1DM [114].

EDs may hasten the development of Type 1 Diabetes Mellitus (T1DM) through several mechanisms, including influencing immune modulation, altering gut microbiota, and affecting the vitamin D pathway [115]. These mechanisms can be briefly summarized as follows:

- *Immunomodulation:* Given the connection between the endocrine and immune systems, the immune system is a primary target of endocrine disruptors (EDs). Exposure to EDs can disrupt immune function and cytokine levels, potentially leading to autoimmune diseases such as T1DM [116].
- *Microbiota:* EDs can metabolized differently by the intestinal microbiota, affecting their absorption, distribution, metabolism, and excretion, which may lead to varying health outcomes [117]. It is proposed that ED exposure could disrupt normal gut microbiota and potentially increase the risk of developing Type 1 Diabetes Mellitus (T1DM). However, the exact mechanisms behind these effects need further investigation [112].
- *Vitamin D:* Vitamin D deficiency is linked to a higher risk of autoimmune diseases, including Type 1 Diabetes Mellitus (T1DM). Vitamin D may offer protection against T1DM by influencing insulin suppression through alterations in CD8 + and CD4 + T cells, B lymphocytes, and macrophage infiltration [118, 119].

#### Bisphenol A (BPA)

 In various in vivo and in vitro studies, it has been determined that BPA has negative effects by binding to nuclear hormone receptors such as estrogenic receptors (Erα, ERβ) or ARs [120–123]. Estrogenic receptors targeted by environmental pollutants such as BPA play a role in glucose metabolism and insulin secretion. Any chemical attack on these receptors may impair glucose homeostasis and the normal physiology of pancreatic cells, implicating the causes of diabetes. Therefore, any chemical attack on these receptors, whether natural or environmental, damages glucose homeostasis and the normal physiology of pancreatic cells [124, 125], as well as inducing insulin resistance by disrupting peripheral insulin receptors or reducing insulin secretion by acting on insulin-secreting pancreatic β cells [126, 127]. Glucagon secreted by pancreatic α-cells has been shown to be substantially impaired by even low doses of BPA exposure [128–130]. Additionally, the ability of BPA to disrupt the biosynthesis and secretion of insulin in β cells has been confirmed by various animal studies [131, 132]. ERs and ARs are regarded as potential therapeutic targets for preventing obesity and metabolic disorders because they play crucial roles in regulating lipid accumulation and enhancing insulin sensitivity [133].

BPA has been shown to affect insulin synthesis and release by pancreatic β cells, as well as disrupt insulin signaling in insulin-sensitive tissues such as the liver, muscle, and fat [134]. esearch indicates that acute BPA exposure leads to temporary hyperinsulinemia, while chronic exposure suppresses adiponectin release and worsens insulin resistance, obesity-related conditions, and diabetes [20, 135]. It has been suggested that prenatal BPA exposure may alter the structure and function of the endocrine pancreas [136] and cause decreased insulin production for up to two generations in adulthood (in rats) [137, 138]. It may also increase insulin resistance, exacerbating T1DM [139]. In another study, prenatal BPA exposure was associated with increased plasma glucose (in male) [140].

In a study of 107 healthy children (ages 8.5–16.1 years), higher urinary BPA was inversely related to insulin resistance [141]. mong 141 obese children (ages 4–16 years), higher urinary BPA was positively correlated with HOMA and negatively correlated with serum adiponectin [142]. In an analysis conducted on children and adolescents with T1DM aged between 3 and 25 years and healthy children of the same age, urinary BPA levels of the T1DM group were significantly higher than the control group [143]. Children with T1DM had significantly higher urinary BPA levels than controls. In a study of 50 T1DM patients (ages 5–18 years), BPA levels were inversely related to birth weight, but no significant link was found between BPA levels and T1DM itself. The small sample size and cross-sectional design limit insights into the mechanisms connecting BPA levels and T1DM [144].

#### Phytalates

 Phthalates have been identified as antagonists of thyroid hormones and androgens, potentially influencing adipogenesis, lipid accumulation, and insulin resistance by modulating the activation of PPARs [145]. Maternal exposure to di(2-ethylhexyl)phthalate (DEHP) significantly impacts metabolic disorders by affecting GLUT2 expression and epigenetic changes in the liver, contributing to insulin resistance in immature male rat offspring [146]. DEHP exposure can disrupt glucose homeostasis in offspring (in rats) through the JAK2/STAT3/SOCS3 pathway, which regulates insulin and leptin signaling [147]. Additionally, DEHP has been reported to cause overexpression of FOXO1, leading to insulin resistance and increased hepatic lipid accumulation (in mice) [148].

One systematic review found that prenatal phthalate exposure was negatively associated with insulin and glucose levels in boys [149]. Earlier reviews and meta-analyses have linked phthalate exposure to a higher risk of diabetes and insulin resistance [150, 151]. However, more studies are needed because most of the evidence is obtained from cross-sectional studies, the number of studies is small, and the relationships of phthalates with insulin and glucose are not consistent [152]. In a recent systematic review, Although the little evidence available on prenatal phthalate exposure and glucose metabolism is somewhat conflicting, results from postnatal assessment have been consistent in showing positive associations of multiple phthalates with fasting blood glucose, insulin levels, and/or HOMA-IR (mostly high-quality reviewed articles according to the results)[70].

However, more studies are needed because most of the evidence is obtained from cross-sectional studies, the number of studies is small, and the relationships of phthalates with insulin and glucose are not consistent [153].

#### Persistent Organic Pollutants (POPs)

 POPs have direct effects on insulin signaling leading to insulin resistance [8]. Although POPs are banned or restricted, these toxic substances can bioaccumulate within the food chain and can still be detected in human tissues worldwide [154].

##### Dichlorodiphenyl trichloroethane (DDT) and Dichlorodiphenyl dichloroethene (DDE)

It has been suggested that DDT and p,p'-DDE may show toxicity through anti-androgenic, estrogenic and anti-estrogenic effects [154]. One animal study suggested that pp,p'-DDT exposure may reduce the secretory activity of the pancreas, which may lead to impaired insulin secretion [155]. It is also thought that p,p'-DDT and p,p'-DDE may disrupt glucose metabolism and possibly induce insulin resistance due to disruptions in lipid homeostasis [156].

No link to diabetes was found in a birth cohort study measuring DDE levels [157]. However, another study showed that 75 children newly diagnosed with Type 1 Diabetes Mellitus (T1DM) had significantly higher serum levels of eight out of nine examined organochlorine and organophosphorus pesticides compared to healthy controls [158]. Additionally, a Swedish case–control study involving 150 children with T1DM and 150 matched controls found that elevated levels of p,p'-DDE in maternal serum during pregnancy were associated with an increased risk of developing T1DM [159].

##### Per- and polyfluoroalkyl substances (PFAS)

 In T1DM, environmental pollutants like PFASs can potentially trigger the autoimmune process affecting β-cells [112]. Specifically, PFOAs and tributyltin (TBT) can disrupt fat storage, adipocyte differentiation, and insulin sensitivity by interfering with the expression of PPARs [154]. High prenatal PFAS exposure has been found to alter lipid profiles in newborns, which may increase the risk of islet autoimmunity and T1DM [114].

he first study to examine serum PFAS concentrations in 25 children and adolescents with Type 1 Diabetes Mellitus (T1DM) found that those newly diagnosed with T1DM had significantly higher PFOS levels compared to matched healthy controls, while PFOA levels were similar. This study suggests that even low levels of PFAS exposure may negatively impact the immune system, potentially increasing the risk of developing T1DM [113]. Conversely, another study found that for children with a high genetic risk of T1DM, low levels of various persistent organic pollutants (POPs), including 14 PFAS, were not associated with β-cell autoimmunity or progression to clinical T1DM [157].

##### Dioxins

 Experimental data show that TCDD can impact glucose regulation at various levels, including β-cell insulin secretion and insulin signaling in metabolically active tissues [160–162]. Since dioxins are widespread, all humans are likely to have some background exposure. Given the high toxicity of dioxins, further human studies are needed to explore the relationship between serum dioxin levels and the development of diabetes mellitus [112].

##### Organophosphorus pesticides (OP)

 Maternal exposure to xenobiotics, such as pesticides, affects both fetal exposure during pregnancy and infant exposure through breastfeeding. Organophosphorus pesticides (OPs) can cross the placenta and have been linked to a prediabetic state in neonates [163]. Exposure to OPs during the prenatal and postnatal periods can disrupt glucose, lipid, and insulin homeostasis, impairing glucose tolerance and insulin sensitivity. This may elevate the risk of developing diabetes and other metabolic disorders, such as Type 2 Diabetes Mellitus, coronary heart disease, or dyslipidemia, in adulthood [164].

### Cardiovascular Diseases

The fetal period is a critical window for sensitivity to the potential impact of environmental factors on the cardiovascular system [165]. Environmental endocrine disruptors in the womb can cause a permanent deterioration in the cardiovascular profile through mechanisms that include epigenetics and endocrine disruption [43, 166]. Additionally, prenatal exposure to low concentrations of EDs has a negative impact on cardio-metabolic risk factors in preschool children [5]. Table 2 shows the effects of endocrine disruptors on the cardiovascular system in children.

**Table 2:** Effects of endocrine disruptors on the cardiovascular system in children

Endocrine disruptors	Health outcomes	Potential mechanisms
Bisphenol A	• Hypertension	• Increasing levels of Ang, Ang-converting enzyme, and angiotensinogen (precursor of Ang) • reducing eNOS levels • Enhancing AngII/ (CaMKII)-α dissociation of eNOS • Increasing ROS/NO imbalance
Phthalates	• Disruption of the renin-angiotensin system • Estrogen and androgens disrupt normal functioning in target organs such as blood vessels and kidneys • Increase in blood pressure	• PPARγ agonist • Competitive binding to sex hormone binding globulin
Persistent organic pollutants	• Disruption of the renin-angiotensin system • Impaired cardiovascular health	• Disruption of sex steroid hormones • Hypothalamic, pituitary adrenal axis disruption • Change in kidney structure and function • Epigenetic modifications • oxidative stress

Ang Anjiyotensin; CaMKII Ca2 + /calmodulin-dependent protein kinase II; eNOS Endothelial nitric oxide; NO Nitric oxide; PPARγ Peroxisome proliferator-activated receptor gamma; ROS Reactive oxygen species.

#### Bisphenol A (BPA)

 BPA exposure raises levels of angiotensin, angiotensin-converting enzyme, and angiotensinogen, which are key for cardiovascular function [167–170].

It also induces hypertension by lowering endothelial nitric oxide (eNOS) levels, promoting the dissociation of eNOS via AngII/Ca2 + /calmodulin-dependent protein kinase II (CaMKII)-α, and increasing the imbalance between vascular reactive oxygen species (ROS) and nitric oxide (NO) [171, 172].

BPA exposure can cross the placenta from the mother to the fetus. However, there is limited epidemiological data linking prenatal BPA exposure to cardiometabolic risk factors in children [40, 140, 173, 174]. Previous studies have reported that prenatal BPA exposure is associated with higher diastolic blood pressure (DBP) [174] or no association in children [40], or with lower DBP in boys and systolic blood pressure (SBP) in girls if prenatal urinary BPA concentrations are above a certain threshold [173].

#### Phytalates

 Prenatal phthalate exposure impacts cardiovascular risk in children with variations based on sex and can be influenced by adolescence [149]. Phthalates, due to their structural similarity to sex hormones, may compete with these hormones for binding to sex hormone-binding globulin during puberty, potentially disrupting the normal function of estrogens and androgens in organs like blood vessels and kidneys [175, 176]. Additionally, because phthalates are PPARγ agonists, they may act through modulation of the renin-angiotensin system [177, 178]. A meta-analysis indicates that prenatal phthalate exposure affects cardiovascular risk in children differently based on sex and is influenced by puberty. During adolescence, physiological and hormonal changes, such as variations in sex hormone secretion, contribute to this effect [179]. Estradiol and testosterone, which are crucial for blood pressure regulation, play significant roles in this process [180, 181]. Phthalates have been shown to increase blood pressure, potentially through mechanisms such as oxidative stress, alterations in cholesterol levels, and inflammatory response deregulation [182]. In animal studies, maternal exposure to DEHP has been linked to increased blood pressure in offspring, potentially due to disruptions in the activation of endothelial nitric oxide synthase and angiotensin II signaling pathways [183].

Exposure to some phthalates during childhood has been associated with lower SBP and, to a lesser extent, lower DBP [174]. In contrast, two cross-sectional studies in children aged 8–19 years reported higher SBP and DBP with higher phthalate exposure [184, 185]. One study reports that exposure to phthalates in childhood is positively associated with SBP, but maternal phthalate exposure is negatively associated with blood pressure in children ages 4 to 11 [182]. Similarly, phthalate exposure during pregnancy has been reported to cause a decrease in SBP at ages 4 and 7 years [186]. One systematic review noted inconsistent findings among previous review articles regarding associations between phthalate exposure and blood pressure/hypertension [149]. Golestanzadeh, Riahi, and Kelishadi (2019) conducted the first systematic review on the topic and found that prenatal phthalate exposure is positively associated with cardiometabolic risk factors in children and adolescents [187].

A recent systematic review found little evidence about the role of phthalates on blood pressure, and results are conflicting. Accordingly, phthalates are associated not only with hypertension but also with abnormally reduced blood pressure levels [70].

Prenatal exposure to phthalates may impact future cardiovascular disease by increasing childhood triglycerides in boys and lowering HDL-C in children, according to a meta-analysis [188]. DEHP exposure reportedly increases blood lipids and triglyceride and total cholesterol accumulation through the LXR/SREBP-1c/PPARα/γ and NF-κB signaling pathways [189]. The link between phthalate exposure and cardiometabolic risk factors in children and adolescents remains debated. A systematic review and meta-analysis of 35 studies—comprising 17 cohort, 15 cross-sectional, and 3 case–control studies—found significant associations between phthalates and measures like BMI, BMI z-score, waist circumference, and levels of low-density lipoprotein cholesterol, triglycerides, and blood glucose. This highlights the need for strategies to prevent phthalate exposure as part of cardiovascular disease prevention efforts [187, 190]. It has been reported that the associations between phthalate exposure and childhood blood pressure and glucose should be monitored further prospectively to investigate whether the associations persist or reverse at later ages [149].

#### Persistent Organic Pollutants (POPs)

 Few studies have directly examined the impact of prenatal POP exposure on cardiovascular health. A review found only two studies on the effect of prenatal POPs on blood pressure in children [191]. One study noted increased systolic or diastolic blood pressure with higher exposure to p,p'-DDE and PCBs in 4-year-olds [78], while another linked higher diastolic blood pressure to PCBs in 7- to 9-year-olds [192]. Conversely, a study found reduced diastolic blood pressure with PCB exposure in children aged 6 to 11 [174]. Some studies found no relationship between POPs or PFASs and blood pressure, though PFOA and PFOS were noted to increase cardiovascular disease risk more than other PFASs [193–195].

A recent review found gender-specific effects on blood pressure: β-hexachlorocyclohexane (β-HCH) and p,p'-DDE were linked to higher systolic blood pressure in girls but not in boys. This gender difference may be due to disruptions in sex steroid hormones, which are crucial for regulating components of the renin-angiotensin system and cardiovascular health. This finding should be confirmed by further studies [196]. Possible biological mechanisms for cardiovascular disease programming include disruption of the hypothalamic–pituitary–adrenal axis, changes in kidney structure and function, epigenetic modifications, and oxidative stress [197].

### Kidney Structure and Function

The literature indicates a lack of sufficient studies on the developmental impact of fetal exposure to endocrine disruptors (EDs) on kidney health, despite its biological plausibility [198]. EDs are thought to cause oxidative stress, enzymatic changes, DNA damage, and direct cytotoxic effects in the kidneys [199]. Observational studies in children with chronic kidney disease have not shown a direct link between BPA and phthalates with clinical outcomes but have found associations with increased biomarkers of tubular damage and oxidative stress [200, 201].

Most studies in children and adolescents are cross-sectional and show an increase in the albumin/creatinine ratio with higher BPA and DEHP levels. However, findings on albuminuria are inconsistent [202–204].

Tsai et al. (2016) studied 184 children under 10 who had high DEHP exposure from consuming certain foods. They found a significant association between DEHP exposure and an increased albumin/creatinine ratio. Children in the high exposure group had a 10.4-fold higher risk of microalbuminuria compared to those with lower exposure [202]. In children with chronic kidney disease, levels of phthalate ester metabolites were lower compared to controls. Some phthalate esters showed negative correlations with the urine protein/creatinine ratio, while others had positive correlations with the estimated glomerular filtration rate [201]. Given the variability in the results of studies, it is not currently possible to speak of a clear and consistent relationship between phthalate esters and children with kidney disease.

In children with high exposure levels, serum PFOS, PFOA, and perfluorohexanesulfonate were cross-sectionally linked to markers of renal function, such as estimated glomerular filtration rate and serum uric acid. However, no prospective associations were observed [205–207].

## Nutrition-Based Approaches to Reducing EDC Exposure

Parents play an essential role in influencing what they and their children are exposed to. Therefore, the most effective step to minimize exposure to EDCs is the family environment. For infants and young children, breastfeeding and then using pureed fresh foods for children, avoiding bottle feeding, and processed foods such as purees effectively reduce EDC exposure. In addition, in the general population, avoiding canned foods and drinks containing plastic, and when these cannot be avoided, using unpackaged foods, cardboard packaging, glass or stainless steel food containers, or drink bottles instead of plastic is effective in reducing EDC exposure. It is also recommended that the consumption of organic foods be encouraged and that fast food or processed foods be avoided [208]. While some studies have shown that dietary interventions effectively reduce EDC levels in the body [209, 209, 210], others have found no effect [211, 212]. In effective studies, urinary EDC concentrations were significantly reduced when participants consumed low-EDC foods or were directed to fresh foods. Consumption of canned foods, in particular, increased EDC levels [213]; while reducing the intake of calories, fat, and processed foods effectively reduced these levels [209, 209, 210]. However, some studies have shown that fresh food consumption does not change EDC levels [211, 212].

**Fig. 2:**
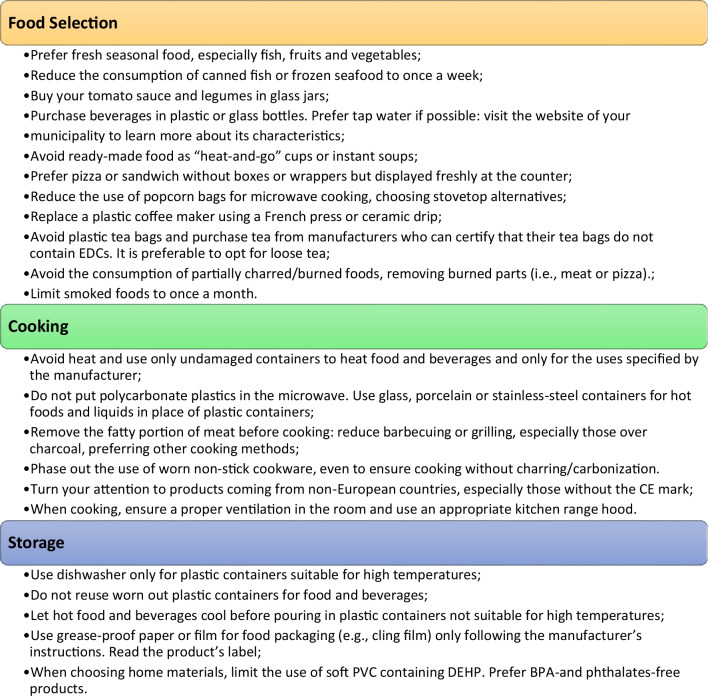
Practical arrangements for food selection, cooking and storage to reduce EDC exposure [6]

Figure 2 includes practical regulations for food selection, cooking, and storage, prepared by the National Institute of Health [214] and integrated with some other epidemiological findings [215–217] by Rolfo et al. (2020) [6].

Such individual precautions against endocrine disruptors (EDCs) can be difficult to identify all exposure sources because it is impossible to know which chemicals are used in many products by individuals [217, 218]. Health professionals should be aware that endocrine disruptors pose serious risks to prenatal and postnatal development and should be guided in implementing effective strategies to reduce these risks and make environmental health a part of medical education [219].

## Conclusion

In this review, we summarized some EDCs such as bisphenol A, phthalates and POPs that negatively affect obesity and metabolic disorders in the pediatric population. Table 3 summarizes several studies related to endocrine disruptors in child obesity and associated disorders. Although the overall evidence for the pathogenetic role of EDCs appears convincing, data on prenatal or postnatal exposure are insufficient and it is not easy to draw definitive conclusions. However, it has been determined that EDCs can disrupt critical biological processes in both prenatal and postnatal periods and cause long-term health problems. In particular, by affecting adipogenesis, energy homeostasis, and hormone signaling, they can increase the risk of obesity, diabetes, and metabolic syndrome. Given these long-term effects, identifying these EDCs and their sources is crucial to preventing childhood obesity and other metabolic disorders. Prospective human studies are needed to understand the consequences associated with other metabolic effects, including the obesogenic and diabetogenic effect of EDCs on metabolic development.

**Table 3:** Studies on the effects of some endocrine disruptors on obesity and related metabolic diseases

Author, year	Study population	EDC	Results
Obesıty			
Valvi et al.,2013 [27]	Spanish birth cohort study (n = 402)	Prenatal BPA exposure	For an association between BPA exposure and obesity-related outcomes at the age of 4 years, although not in infancy
Harley et al.,2013 [29]	Center for the Health Assessment of Mothers and Children of Salinas (CHAMACOS) 498 pregnant women and 402 children	Prenatal and postnatal BPA Exposure	At 9 years of age, higher urinary BPA concentrations were linked to increased adiposity. Conversely, higher BPA levels in mothers during pregnancy were associated with lower BMI, body fat, and reduced risk of overweight/obesity in their daughters at the same age
Braun et al., 2014 [28]	297 mother–child pairs from Cincinnati, Ohio (HOME Study)	Prenatal and early-childhood BPA exposures	Prenatal and early-childhood BPA exposures (maternal urine) were not associated with increased BMI at 2–5 years of age, but higher early-childhood BPA exposures were associated with accelerated growth during this period
Junge et al., 2018 [30]	LINA mother–child-study obtained during pregnancy (n = 552)	Prenatal BPA exposure	Prenatal BPA exposure seems to be a contributing factor in the development of an early overweight phenotype
Braun et al., 2019 [31]	MIREC Study, a prospective Pan-Canadian pregnancy and birth cohort study (n = 719)	Gestational BPA exposure	Gestational urinary BPA concentrations were associated with subtle increases in girl's central adiposity during early childhood
Jacobson et al., 2019 [42]	NHANES 6–19 years, 1831 children and adolescents	BPA exposure	BPA and total bisphenols were not statistically significantly associated with general obesity, abdominal obesity, or any body mass outcome
Guo et al., 2024 [32]	Systematic review (included thirteen studies, with participant counts ranging from 173 to 1124 mother–child dyads)	Prenatal BPA exposure	potential effects of prenatal BPA exposure on offspring obesity (such as BMI z-score, WC, overweight/obesity, skinfold thickness, body fat (%))
Boas et al., 2010 [53]	845 children 4–9 years of age	Childhood phthalates exposure	Phthalate metabolites were negatively associated with height, weight, body surface, and height gain in both sexes
Vafeiadi et al., 2016 [40]	500 mother–child pairs from the RHEA pregnancy cohort	Early life exposure to BPA	Higher urinary BPA concentrations in children at age 4 were linked to increased BMI z-scores, waist circumference, and skinfold thickness. In contrast, BPA levels during early pregnancy were associated with lower BMI and adiposity in girls
Shoaff et al., 2017 [65]	219 mother–child pairs	Early-Life Phthalate Exposure	The results suggest that the associations between child adiposity at 8 y of age and urinary MEP and ∑DEHP concentrations depended on timing of exposure. In addition, urinary MBzP concentrations during pregnancy and childhood were inversely associated with adiposity
Gao et al., 2022 [54]	Systematic review (The combined subjects of the 14 studies were 10,396 mother–child pairs)	Prenatal phthalate exposure	Current articles did not find a relationship between prenatal phthalate exposure and age-specific outcomes in children, except for positive associations between prenatal MEP exposure and absolute adiposity markers. Nonetheless, epidemiological data suggested a weak, sex-specific link between prenatal phthalate exposure and children's obesity trajectory
Guo et al., 2020 [56]	251 mother–infant pairs	In-utero exposure to phthalates	Some gender-specific associations of meconium exposure to phthalates with some parameters of birth size
Lee et al., 2022 [64]	Systematic review and meta-analysis (including 22 longitudinal and 17 cross-sectional studies)	Prenatal phthalate exposure	Prenatal exposure to phthalates was found to be associated with decreased BMI z-score in children, but not associated with body fat percentage
Perez-Diaz et al., 2024 [70]	Systematic review (including 58 articles)	Prenatal and postnatal phthalate exposure	The evidence revealed a positive association between prenatal (in utero) exposure to most phthalates and markers of obesity in the offspring, but contradictory results when postnatal exposure and obesity were assessed
Valvi et al., 2012 [75]	344 children from a Spanish birth cohort	Prenatal Concentrations of Polychlorinated Biphenyls, DDE, and DDT	Prenatal PCB, DDE, and DDT exposures may be associated with overweight in children and that sex and high-fat intake may influence susceptibility
Iszatt et al., 2015 [85]	European birth cohorts with biomarker concentrations of polychlorinated biphenyl 153 (PCB-153) (n = 2.487), and p,p´-dichlorodiphenyldichloroethylene (p,p´-DDE) (n = 1.864)	Prenatal and Postnatal Exposure to Persistent Organic Pollutants	In a large and heterogeneous European population, we found an increase in infant growth associated with prenatal p,p´-DDE and a decrease associated with postnatal PCB-153 exposure
Stratakis et al., 2022 [72]	Systematic review (33 studies reporting associations with prenatal organochlorine exposure, 21 studies reporting associations with prenatal PFAS, and five studies reporting associations with prenatal PBDEs)	Prenatal persistent organic pollutants exposure	No significant associations between PCB-153, PFOA, PFOS, or pentaPBDEs with childhood BMI were found in meta-analyses. In individual studies, there was inconclusive evidence that POP levels were positively associated with other obesity indicators (e.g., waist circumference)
Tang-Péronard et al., 2014 [74]	656 pregnant women	Prenatal polychlorinated biphenyl exposure	Prenatal exposure to PCB and DDE may play a role for subsequent obesity development. Girls whose mothers have a high prepregnancy BMI seem most affected
Dıabetes mellıtus			
Menale et al., 2017 [142]	141 obese children	BPA exposure	Urinary BPA levels are directly associated with insulin resistance regardless of BMI
Carlsson et al., 2018 [141]	Copenhagen Puberty Study, 107 healthy normal-weight children	BPA, phthalate metabolites	Healthy normal-weight children suggests an inverse association between BPA and insulin resistance
İnce et al., 2018 [144]	5 and 18 years including 50 children with T1DM and 50 healthy children	BPA exposure	There was no significant association between urinary BPA levels and T1DM, we found an inverse relationship between current urinary BPA levels and birth weight
Ouyang et al., 2020 [140]	218 pregnant women	Prenatal BPA exposure	Medium prenatal BPA levels were linked to higher plasma glucose in boys, while maternal prenatal urinary BPA concentrations were associated with higher SBP and DBP in girls, but not in boys, independent of adiposity
Tosirisuk et al., 2022 [143]	75 T1DM children and adolescents and 113 age-matched controls	BPA exposure	Higher urinary BPA level is one of the possible risk factors for T1DM
Castro-Correia et al., 2018 [153]	302 T1DM patients	Phthalate exposure	No statistically significant differences were found in the concentrations of urinary metabolites of DiBP and DBP among newly diagnosed diabetic children, children with established type 1 diabetes and healthy controls. However, children recently diagnosed with diabetes showed a tendency to have higher DiBP metabolite concentrations
Zhang et al., 2022 [150]	Systematic review and meta-analysis (24 articles were retained for inclusion, 7 of which evaluated the association between phthalate exposure and DM, 20 studies were selected for systematic review on the effects of phthalate on insulin resistance)	Phthalate exposure	Exposure to phthalates, especially MMP, MnBP, MiBP, MCPP and DEHP metabolites, might be a risk factor of DM. Our results should be interpreted with caution due to heterogeneous design of enrolled studies
Gao et al., 2023 [149]	Systematic review and a pilot meta-analysis (included studies. Overall, a total of 6074 children, including 2745 girls and 3325 boys)	Prenatal phthalate exposure	Prenatal phthalate exposure interfered with cardiovascular risk in children with gender-specific differences and was influenced by puberty. Overall, prenatal ∑DEHP was negatively associated with systolic blood pressure in girls and with insulin and glucose in boys but increased the level of triglycerides
Perez-Diaz et al., 2024 [70]	Systematic review (58 articles)	Prenatal and postanatal exposure	The evidence so far shows a positive overall association of prenatal phthalate exposure with obesity, and postnatal exposure with markers of diabetes and less consistently, with TG levels
Rignell-Hydbom et al., 2010 [159]	150 cases (children who had their diagnosis mostly before 18 years of age) and 150 controls,	Prenatal POPs exposure	In utero exposure to POPs will trigger the risk for developing type 1 diabetes was not supported by the results. The risk estimates did, although not statistically significant, go in the opposite direction
Cardıovascular dıseases			
Bae et al., 2017 [173]	645 children (the age of 4)	Prenatal BPA exposure	The present study suggests that exposure to BPA during pregnancy is associated with higher diastolic BP of the children above a certain threshold
Warembourg et al., 2019 [174]	1.277 children from the European HELIX	Prenatal BPA, phthalates, perfluoroalkyl substances (PFAS)	This study suggests that early-life exposure to several chemicals, as well as built environment and meteorological factors, may affect BP in children
Trasande et al., 2013 [185]	2838 children aged 6–19 years	Phthalate exposure	Dietary phthalate exposure is associated with higher systolic BP in children and adolescents
Trasande et al., 2015 [184]	1619 participants, 6–19 years	Phthalate exposure	A significant association of di-isononyl phthalate (DINP) and di-isodecyl phthalate (DIDP) metabolites, currently used as DEHP replacements, with higher SBP
Valvi et al., 2015 [186]	First and third pregnancy trimesters (n = 391)	Prenatal phthalate exposure	Prenatal phthalate exposure may be associated with postnatal growth and blood pressure in a sex-specific manner. Prenatal exposure to phthalates may influence postnatal growth and blood pressure differently in boys and girls up to 7 years of age
Lee et al., 2016 [192]	214 children (7–9 years)	POPs exposure	Low-dose exposures to PCBs among children in the general population could negatively influence metabolic health, particularly diastolic BP
Geiger et al., 2014 [194]	815 participants ⩽18 years of age	PFOA abd PFOS exposure	Serum PFOA and PFOS are significantly associated with dyslipidemia in adolescents, even at the lower “background” exposure levels of the US general population
Abdullah Soheimi et al., 2021 [195]	Meta-Analysis (29 articles)	POPs exposure	Total cholesterol levels in serum lipids are often associated with PFC exposure, with perfluorooctanoic acid (PFOA) and perfluorooctane sulfonic acid (PFOS) linked to a higher risk of cardiovascular diseases compared to other PFCs
Rouxel et al., 2023 [196]	1667 mother–child pairs	Prenatal exposure to multiple POPs	This study indicates that prenatal exposure to background levels of POPs, especially organochlorine pesticides, is linked to adiposity markers up to age 12 in both genders, with HCB being the main contributor. Additionally, β-HCH and p,p′-DDE were associated with higher systolic blood pressure in girls, while no significant links were found for PCBs
Kıdney structure and functıon			
Jacobson et al., 2020 [200]	618 children and adolescents	BPA and phthalate exposure	While BPA and phthalate metabolites were not linked to clinical renal endpoints like eGFR or proteinuria, there was a consistent pattern of increased tubular injury and oxidative stress over time, potentially impacting long-term renal function
Malits et al., 2018 [201]	1 to 17 years old (n = 538)	BPA and phthalate exposure	Urinary excretion of BPA and phthalates is lower than in the general population. For certain phthalate metabolites, eGFR showed a direct relationship, while the urinary protein-to-creatinine ratio showed an inverse relationship; however, no links were found with blood pressure
Trasande et al., 2014 [203]	667 children	Phthalate exposure	Graded exposure to phthalates is associated with increased risk of low-grade albuminuria in healthy children and adolescents
Tsai et al., 2016 [202]	184 children (≤ 10 yeasrs)	Phthalate exposure	Higher DEHP exposure to DEHP-tainted foods was significantly associated with increase of urine albumin/creatinine ratio
Geiger et al., 2013 [205]	1.772 participants ≤ 18 years of age	Perfluoroalkyl exposure	Serum PFC levels were positively associated with hyperuricemia in a representative, multiethnic sample of US children
Kataria et al., 2015 [207]	1960 participants aged 12–19 years of the	Perfluoroalkyl exposure	PFAAs are associated with a reduction in kidney function and increased uric acid levels in otherwise healthy adolescents

BPA Bisphenol A; DEHP Di(2-ethylhexyl) phthalate; DBP Diastolic blood pressure; HCBHexachlorobenzene; PFAA Perfluoroalkyl and Polyfluoroalkyl Substance; POPs Persistent Organic Pollutants; PCB Polychlorinated Biphenyls; PFOA Perfluorooctanoic Acid; PFOS Perfluorooctane Sulfonate; PFC Perfluorinated Chemicals; SBP Systolic blood pressure.

In this context, it is a fact that strategies to reduce EDC exposure are of critical importance, especially during pregnancy and breastfeeding. Reducing the use of products containing EDC, turning to organic foods, and minimizing environmental pollution is important to reduce exposure. In addition, developing public health policies can effectively limit exposure to these substances. In particular, limiting exposure to these chemicals should be a priority for vulnerable populations such as pregnant women, infants, and young children. To reduce EDC exposure, it is important for the state and health authorities to develop policies on this issue and implement protective interventions for risk groups and individual awareness.

## Data Availability

No datasets were generated or analysed during the current study.
